# Plant-derived nutrition and perimenopausal emotional health: current evidence and research priorities

**DOI:** 10.3389/fnut.2026.1961915

**Published:** 2026-10-19

**Authors:** Zhirui Sun, Xue Liu, Shuyue Dong, Qun Liu, Kun Wu, Xinyue Zhang, Zi Ye, Yi Wen, Maoya Li, Shaobin Wei

**Affiliations:** Department of Gynecology, Hospital of Chengdu University of Traditional Chinese Medicine, Chengdu, China

**Keywords:** perimenopause, emotional health, plant-derived foods, dietary bioactives, botanical preparations, dietary supplements, gut-brain axis, precision nutrition

## Abstract

Perimenopause combines ovarian hormone variability with changes in stress regulation, inflammatory-redox balance, neuroplasticity, and gut-brain metabolism, creating a period of increased vulnerability to depressive symptoms, anxiety, irritability, and sleep disturbance. This narrative review synthesizes evidence on plant-derived foods, bioactives, dietary supplements, and standardized botanical preparations relevant to emotional health during the menopausal transition. Targeted searches of PubMed/MEDLINE, Web of Science, and Scopus identified literature published through 31 July 2026. Evidence was classified by consumed form and reproductive-stage directness and integrated across three regulatory axes: endocrine-stress regulation, immune-redox-neuroplasticity, and gut-brain-metabolic communication. Human evidence includes small randomized trials of standardized saffron, rhapontic rhubarb, fenugreek, ashwagandha, and shatavari preparations, with product-specific psychological-symptom signals, as well as a null Hypericum-Vitex trial. Evidence for plant-rich dietary patterns and isolated bioactives derives mainly from broader menopausal or adult populations. Across studies, reproductive stage, exposure composition, symptom endpoint, and preparation standardization emerge as key determinants of interpretation. Linking symptom clusters with proposed pathway involvement generates hypotheses for prospective phenotype-stratified nutrition trials. Future studies should combine reproductive staging using the Stages of Reproductive Aging Workshop + 10 (STRAW+10) framework, prespecified emotional outcomes, dietary assessment, standardized product characterization, exposure biomarkers, metabotype profiling, and safety monitoring. This framework positions plant-derived nutrition as a testable component of perimenopausal emotional-health research and functional-food development.

## 1 Introduction

Perimenopause is a period of marked ovarian hormone variability and increased vulnerability to depressive symptoms, anxiety, irritability, and sleep disturbance. In the Study of Women’s Health Across the Nation, 23% of 3,302 women aged 42–52 years had elevated depressive-symptom scores at baseline (1), and the likelihood of major depressive episodes increased during peri- and early postmenopause (2). Emotional symptoms commonly cluster with vasomotor symptoms, fatigue, disrupted sleep, and cognitive complaints (3–7), while genitourinary, body-composition, skeletal, and cardiovascular changes form the broader clinical setting (8–12). This clustering reflects coordinated changes across endocrine, neural, immune, and metabolic systems.

Perimenopausal emotional vulnerability arises from the interaction of ovarian-steroid fluctuations with neuroendocrine feedback, stress responsivity, inflammatory-redox signaling, neurotransmission, and gut microbial metabolism (13–21). Plant-derived nutrition is especially relevant because it can be studied at both dietary-pattern and defined-product levels. In whole foods, the surrounding matrix shapes the absorption and microbial conversion of bioactive constituents (22), whereas standardized supplements and botanical preparations allow dose and composition to be specified but require rigorous product identification and quality control (23). Mediterranean and related plant-rich patterns provide combinations of fibers, polyphenols, phytoestrogens, unsaturated fats, and fermented-food substrates within habitual meals (24, 25). This continuum permits analysis of both repeated food-matrix exposures and dose-specific effects of defined preparations.

Human research now spans prospective dietary studies (26, 27), perimenopause-specific trials of standardized botanical preparations (28–33), and systematic reviews of nutritional and plant-derived interventions (34, 35). These evidence streams answer different questions. Dietary studies capture combined exposures but rarely isolate emotional outcomes in well-staged perimenopausal cohorts, whereas product trials test defined preparations but are generally small and short. The central unresolved issue is which plant-derived exposures have evidence relevant to perimenopausal emotional health, in what consumed form, and with what degree of clinical directness. Building on these syntheses, the present review jointly considers consumed form, reproductive-stage directness, and mechanistic rationale to assess the relevance of existing evidence to perimenopausal emotional health (34, 35). This integrated interpretation distinguishes product-specific clinical findings from evidence extrapolated from adjacent populations or experimental models, and identifies questions requiring dedicated perimenopausal trials. The three-axis framework and symptom–exposure mappings are used to organize evidence and generate hypotheses that require prospective validation.

This narrative review examines plant-derived foods, food-derived bioactives, dietary supplements, and standardized botanical preparations in relation to emotional health during perimenopause. It classifies evidence by consumed form and reproductive-stage directness, integrates biological findings across the endocrine-stress, immune-redox-neuroplasticity, and gut-brain-metabolic axes, and uses symptom profiles to formulate hypotheses for perimenopause-specific nutrition trials and functional-food development (Figure 1).

**Figure 1:**
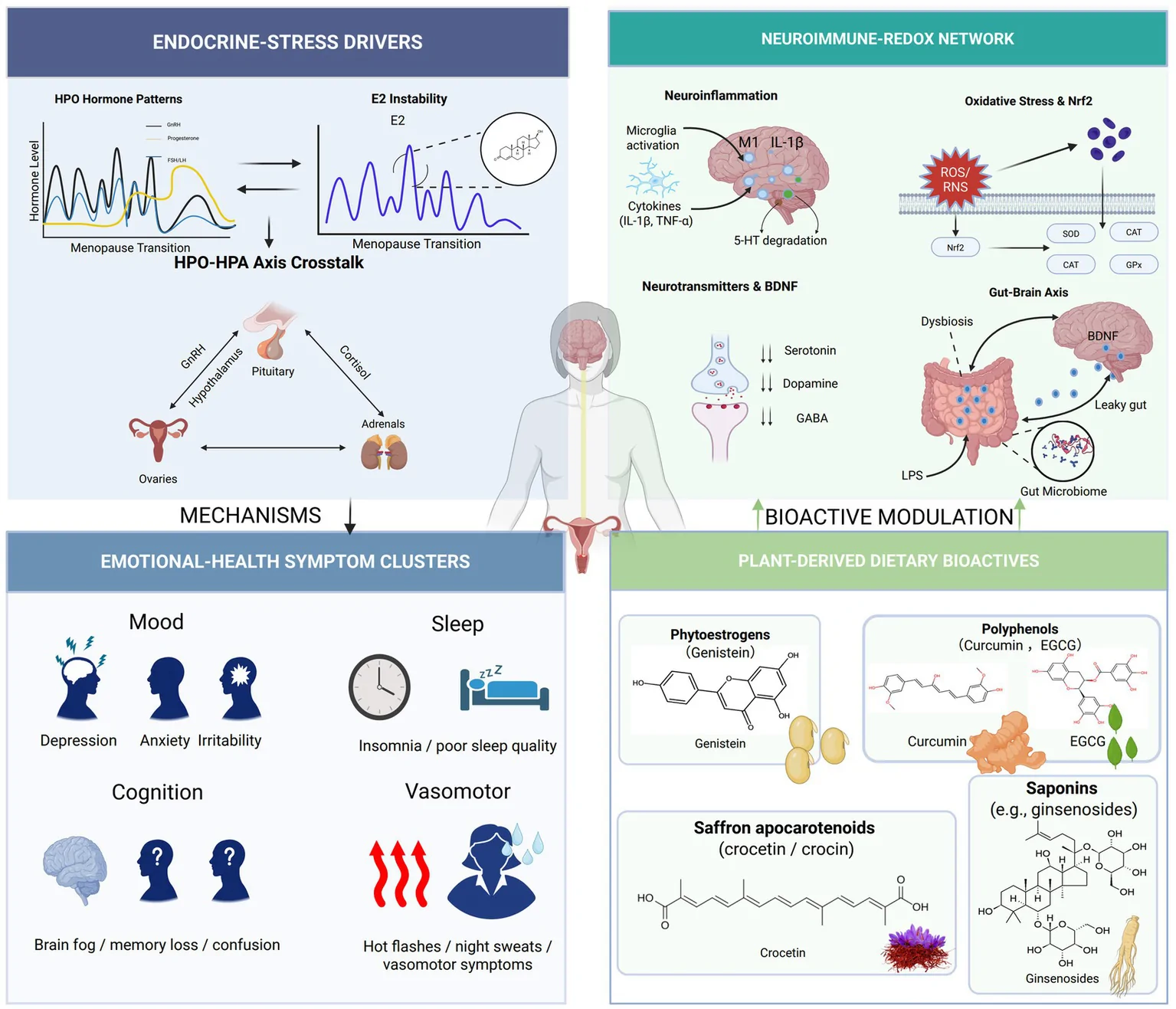
Conceptual overview of plant-derived dietary exposures and emotional-health vulnerability during perimenopause. The schematic summarizes endocrine-stress drivers, neuroimmune-redox and gut-brain-related network changes, symptom clusters, and representative bioactive classes. The compounds shown may be consumed in foods or delivered as isolated or standardized preparations; exposure category and evidence directness are specified in Table 1. 5-HT, 5-hydroxytryptamine (serotonin); BDNF, brain-derived neurotrophic factor; CAT, catalase; E2, estradiol; EGCG, epigallocatechin gallate; FSH, follicle-stimulating hormone; GABA, gamma-aminobutyric acid; GnRH, gonadotropin-releasing hormone; GPx, glutathione peroxidase; HPA, hypothalamic–pituitary–adrenal; HPO, hypothalamic–pituitary-ovarian; IL-1β, interleukin-1β; LH, luteinizing hormone; LPS, lipopolysaccharide; M1, pro-inflammatory microglial phenotype; Nrf2, nuclear factor erythroid 2-related factor 2; RNS, reactive nitrogen species; ROS, reactive oxygen species; SOD, superoxide dismutase; TNF-α, tumor necrosis factor-α. (Created by BioRender.com).

## 2 Methods

For this narrative review, PubMed/MEDLINE, Web of Science Core Collection, and Scopus were searched using three concept groups: reproductive stage (perimenopause, menopause, and postmenopause); plant-derived exposures (dietary patterns, foods, bioactives, supplements, and botanical preparations); and emotional or related outcomes (depression, anxiety, mood, sleep, quality of life, and menopausal symptoms). Synonyms were combined with OR and concept groups with AND. PubMed used Medical Subject Headings and title/abstract terms, Web of Science used Topic fields, and Scopus used title, abstract, and keyword fields. With publication dates limited to 31 July 2026 or earlier and no language or study-design filters, the searches yielded 2,722, 1,977, and 5,152 records, respectively.

Literature was considered for its clinical or mechanistic relevance, prioritizing controlled human studies in perimenopausal women. Targeted searches and reference-list screening provided additional physiological, mechanistic, and safety evidence. Complex individualized multi-herb prescriptions were excluded; fixed-composition botanical combinations were considered as distinct products.

Evidence was classified by consumed form and population directness (perimenopausal, adjacent menopausal or postmenopausal, broader-adult, and preclinical). Study design, sample size, outcome prespecification, attrition, preparation standardization, conflict-of-interest disclosures, and independent replication were considered during synthesis. Findings were synthesized narratively owing to heterogeneity in populations, preparations, and outcomes.

## 3 Three regulatory axes of emotional vulnerability during perimenopause

### 3.1 Endocrine-stress axis: ovarian-steroid variability and stress responsivity

Three interacting regulatory systems connect ovarian steroid variability with emotional symptoms during perimenopause: endocrine-stress regulation, immune-redox-neuroplasticity, and gut-brain-metabolic communication. Their reciprocal interactions provide the mechanistic structure for examining plant-derived dietary exposures in the sections that follow (Figures 2, 3). Perimenopause is characterized by pronounced hormonal variability rather than a uniformly hypoestrogenic state. As ovarian reserve declines, estradiol fluctuates, inhibin B falls, follicle-stimulating hormone rises, and anovulatory cycles become more frequent (13, 14). Greater within-woman estradiol variability has been associated with a higher depressive-symptom burden, and mood responsivity to estradiol differs between perimenopausal and postmenopausal women (16, 17). Changes in hypothalamic–pituitary-ovarian (HPO) feedback also interact with hypothalamic–pituitary–adrenal (HPA) regulation, linking reproductive aging with stress responsivity (18, 36). Estradiol modulates glucocorticoid feedback and corticotropin-releasing hormone signaling in a receptor-, region-, and context-dependent manner, and human studies indicate that estrogen can alter cortisol and catecholamine responses to psychological stress (37–39). Midlife studies have reported late-transition increases in cortisol, flatter diurnal slopes associated with depressive symptoms, and variable cortisol-awakening and laboratory-stress responses (36, 40–42). Longitudinal cohorts consistently identify the menopausal transition as a period of increased vulnerability to depressive symptoms and major depression (1, 2). HPA regulation therefore represents a variable component of this vulnerability, with its contribution likely differing across women and stages (36, 41).

**Figure 2:**
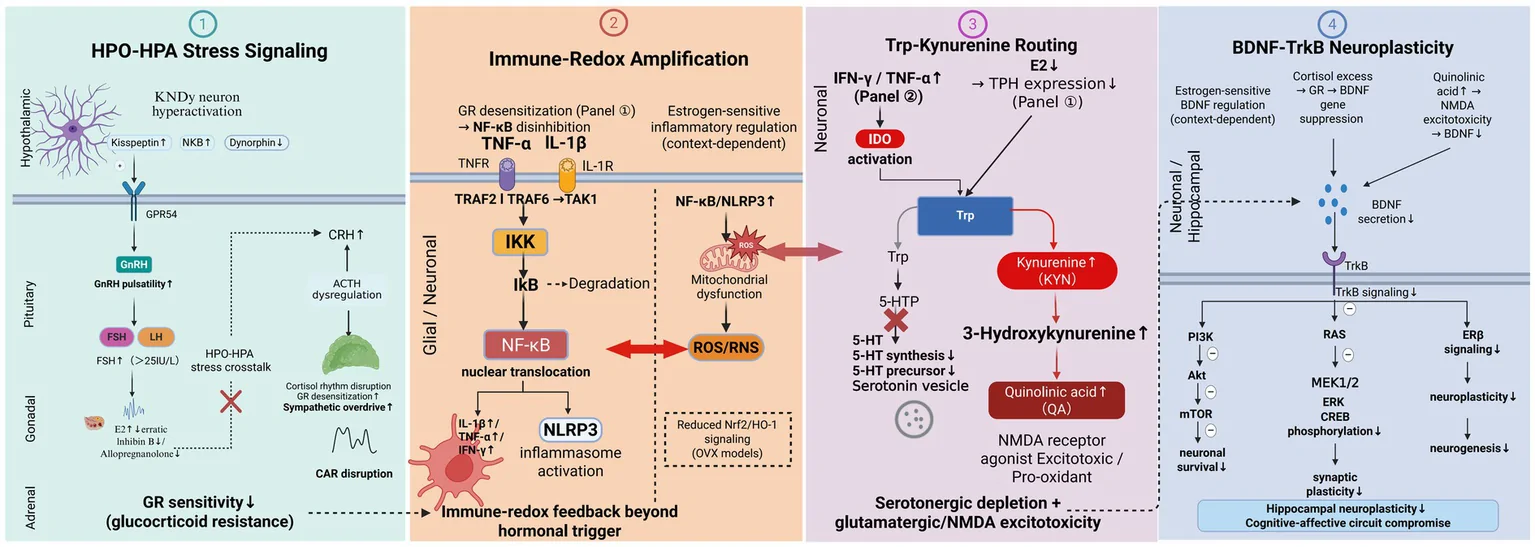
Core mechanisms linking perimenopausal endocrine-stress changes to emotional-health vulnerability. Panels 1–4 show HPO-HPA stress signaling, immune-redox amplification, tryptophan-kynurenine routing, and BDNF–TrkB-related neuroplasticity, respectively. Gut-brain-metabolic communication is shown separately in Figure 3. HPO, hypothalamic–pituitary-ovarian; HPA, hypothalamic–pituitary–adrenal; KNDy, kisspeptin/neurokinin B/dynorphin neurons; NKB, neurokinin B; GPR54, G protein-coupled receptor 54; GnRH, gonadotropin-releasing hormone; FSH, follicle-stimulating hormone; LH, luteinizing hormone; E2, estradiol; CRH, corticotropin-releasing hormone; ACTH, adrenocorticotropic hormone; GR, glucocorticoid receptor; CAR, cortisol awakening response; ERβ, estrogen receptor-β; NF-κB, nuclear factor-κB; TNF-α, tumor necrosis factor-α; IL-1β, interleukin-1β; TNFR, tumor necrosis factor receptor; IL-1R, interleukin-1 receptor; TRAF2/6, tumor necrosis factor receptor-associated factors 2 and 6; TAK1, transforming growth factor β-activated kinase 1; IKK, IκB kinase; IκB, inhibitor of nuclear factor κB; NLRP3, NOD-, LRR- and pyrin domain-containing protein 3; ROS, reactive oxygen species; RNS, reactive nitrogen species; IFN-γ, interferon γ; Nrf2, nuclear factor erythroid 2-related factor 2; HO-1, heme oxygenase-1; OVX, ovariectomized; Trp, tryptophan; TPH, tryptophan hydroxylase; IDO, indoleamine 2,3-dioxygenase; 5-HTP, 5-hydroxytryptophan; 5-HT, 5-hydroxytryptamine (serotonin); KYN, kynurenine; QA, quinolinic acid; NMDA, N-methyl-D-aspartate; BDNF, brain-derived neurotrophic factor; TrkB, tropomyosin receptor kinase B; PI3K, phosphoinositide 3-kinase; Akt, protein kinase B; mTOR, mechanistic target of rapamycin; RAS, Ras-family small guanosine triphosphatases; MEK1/2, mitogen-activated protein kinase kinases 1 and 2; ERK, extracellular signal-regulated kinase; CREB, cyclic adenosine monophosphate response element-binding protein. (Created by BioRender.com).

**Figure 3:**
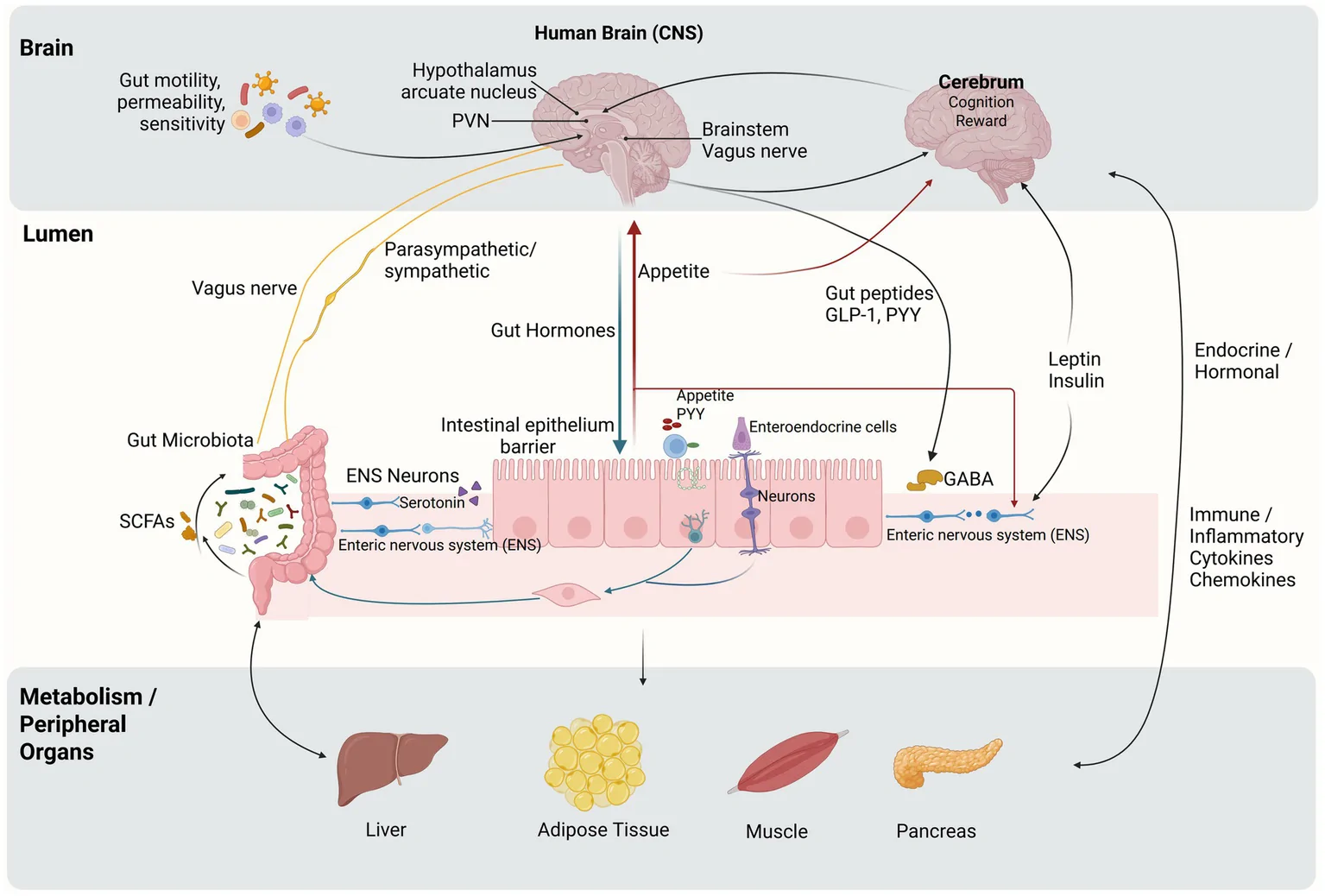
Gut-brain-metabolic communication. Neural, hormonal and immune signals link the gut microbiota and intestinal barrier with the brain and peripheral metabolic organs. CNS, central nervous system; ENS, enteric nervous system; GABA, gamma-aminobutyric acid; GLP-1, glucagon-like peptide-1; PVN, paraventricular nucleus; PYY, peptide YY; SCFAs, short-chain fatty acids. (Created by BioRender.com).

Altered cortisol rhythmicity links the endocrine-stress axis with sleep, cognition, and immune regulation. Dysrhythmic cortisol is associated with disturbed sleep continuity and architecture (7, 43), while prolonged glucocorticoid exposure can impair hippocampal synaptic plasticity and adult neurogenesis (44, 45). These effects intersect with estrogen-sensitive hippocampal circuits and may contribute to cognitive complaints during the menopausal transition (5, 46, 47). Reduced glucocorticoid sensitivity can also sustain pro-inflammatory signaling, connecting endocrine instability with the immune-redox processes described below (48, 49) (Figure 2, panel 1).

### 3.2 Immune-redox-neuroplasticity axis

Estradiol modulates central immune activity by restraining microglial activation and pro-inflammatory transcriptional programs and by supporting blood–brain barrier integrity (19, 50, 51). Variable estrogen signaling during perimenopause may weaken this immunomodulatory restraint and favor a low-grade pro-inflammatory milieu in susceptible women (19). Sustained or dysrhythmic glucocorticoid exposure can further reduce glucocorticoid sensitivity and facilitate cytokine production (48, 49). The convergence of ovarian-steroid variability and altered stress regulation thus provides two interacting inputs to neuroimmune activation. Neuroinflammation and oxidative stress form a bidirectional amplification loop within this axis (20, 52). Mitochondrial dysfunction and reactive oxygen species accumulation activate nuclear factor-κB (NF-κB)-linked signaling and the NOD-, LRR- and pyrin domain-containing protein 3 (NLRP3) inflammasome, promoting caspase-1-dependent release of interleukin-1β (IL-1β) and interleukin-18 (IL-18) and further microglial activation (52). Reduced antioxidant defenses during ovarian-hormone loss can intensify this cycle (20). SWAN analyses showing persistent vasomotor and depressive-symptom trajectories in vulnerable subgroups are consistent with biological processes that extend into early postmenopause (53, 54).

Ovariectomized rodent models provide experimental support for this coupling. These models show hippocampal NF-κB activation, increased IL-1β, lipid peroxidation, and suppression of nuclear factor erythroid 2-related factor 2 (Nrf2)-linked antioxidant programs; estradiol replacement or plant-derived compounds that engage the Nrf2/heme oxygenase-1 (HO-1) pathway can attenuate selected abnormalities (55, 56) (Figure 2, panel 2). In longitudinal human data, high-sensitivity C-reactive protein (hsCRP) and interleukin-6 (IL-6) follow distinct trajectories across the final menstrual period (57), while multimodal neuroimaging demonstrates stage-related changes in brain structure and glucose metabolism during reproductive aging (58). Together, these findings connect endocrine aging with redox, immune, and bioenergetic remodeling.

Acting as downstream interfaces of the immune-redox-neuroplasticity axis, the endocrine and neuroimmune disturbances outlined above converge on monoaminergic neurotransmitter systems that shape mood regulation, sleep architecture, thermoregulation, and cognitive function (59). Estradiol modulates serotonergic (5-hydroxytryptamine, 5-HT) neurotransmission at multiple levels, including tryptophan hydroxylase-2 expression in dorsal raphe serotonergic neurons, monoamine oxidase (MAO)-mediated monoamine catabolism, serotonin transporter (SERT) expression and availability, and serotonin receptor expression (notably cortical 5-HT2A) (59, 60). The erratic estrogen signaling of perimenopause may therefore destabilize this multi-level serotonergic regulation, contributing to mood lability and disrupted sleep–wake cycling in susceptible individuals (59). With respect to thermoregulation, vasomotor symptoms are closely linked to a narrowed thermoneutral zone and heightened central sympathetic/adrenergic activation; although the specific contribution of serotonin remains debated, monoaminergic fluctuations plausibly interact with hypothalamic thermoregulatory circuitry during the menopausal transition (61). Noradrenergic transmission, including locus coeruleus-linked pathways shown to be estrogen-sensitive in menopause-mimicking models, may undergo parallel destabilization, further narrowing the thermoneutral zone and amplifying arousal and autonomic reactivity, thereby linking vasomotor, sleep, and anxiety symptom domains through shared noradrenergic mechanisms (61, 62).

Neuroinflammation can amplify monoaminergic disruption through tryptophan metabolism (63, 64). Interferon-γ (IFN-γ) and tumor necrosis factor-α (TNF-α) induce indoleamine 2,3-dioxygenase, redirecting tryptophan from serotonin synthesis toward the kynurenine pathway (63). This shift reduces serotonin-precursor availability and increases neuroactive metabolites such as quinolinic acid, an N-methyl-D-aspartate (NMDA)-receptor agonist with excitotoxic and pro-oxidant properties (63, 64). The pathway links inflammatory signaling, serotonergic depletion, and glutamate-related stress (63, 64) (Figure 2, panel 3). GABAergic (γ-aminobutyric acid, GABA) inhibitory neurotransmission, which buffers limbic circuits against stress-related hyperactivation, is simultaneously compromised through complementary mechanisms (65, 66). Allopregnanolone, a potent positive allosteric modulator of GABA-A receptors and a neurosteroid metabolite of progesterone, declines as progesterone production wanes alongside the rising frequency of anovulatory cycles during perimenopause (65, 67). Progesterone-linked neurosteroid withdrawal reduces GABAergic inhibitory tone in limbic circuits, lowering the threshold for the expression of anxiety- and stress-related symptoms (65, 66). Inflammatory cytokines further modulate the surface expression and trafficking of GABA-A receptor subunits, potentially compounding the functional deficit in inhibitory neurotransmission and connecting GABAergic vulnerability back to the immune-redox-neuroplasticity axis (68).

Melatonin links tryptophan metabolism to circadian regulation: in mammals, it is synthesized from serotonin via N-acetylserotonin (69). Nocturnal pineal secretion signals biological night, and altered melatonin timing or levels have been reported in depression, although findings in humans are heterogeneous (70). Experimental studies also support antioxidant and context-dependent immunomodulatory actions, including anti-inflammatory effects under inflammatory conditions (71). Clinical evidence concerns exogenous melatonin: one meta-analysis of randomized trials in menopausal women found no significant benefit for sleep quality, anxiety, or depression (72), whereas a later meta-analysis focused on postmenopausal women reported reductions in depression and anxiety scores (73). These pooled findings should not be interpreted as establishing efficacy for emotional symptoms in specifically staged perimenopausal women.

Brain-derived neurotrophic factor (BDNF) integrates endocrine, stress, and inflammatory signals at a neuroplastic endpoint. Estradiol promotes BDNF expression in hippocampal-prefrontal circuits through estrogen-receptor signaling (74), whereas prolonged glucocorticoid exposure can reduce BDNF-linked trophic support and synaptic plasticity (75). In perimenopausal women, depressive symptoms have been associated with lower serum BDNF and higher inflammatory markers, including TNF-α (76); meta-analytic evidence in major depressive disorder likewise shows lower peripheral BDNF and partial recovery with antidepressant treatment (77). These findings place BDNF-related signaling at the intersection of estrogen variability, HPA activation, and neuroinflammation (74–76), while circulating BDNF remains a clinical correlate rather than a direct measure of central tropomyosin receptor kinase B (TrkB) activity (Figure 2, panel 4).

### 3.3 Gut-brain-metabolic axis

The gut microbiota contributes to neuroendocrine and immune regulation, and its diversity, composition, and hormone-related functions change across the menopausal transition and postmenopause (78, 79). Microbial enzymes also participate in steroid handling (79, 80). The estrobolome, particularly bacterial β-glucuronidases, deconjugates estrogen glucuronides in the gut and facilitates enterohepatic recirculation (80). Changes in community structure and estrogen-reactivation capacity may therefore modify systemic hormone exposure during reproductive aging (78, 80, 81). Gut dysbiosis can amplify neuroimmune signaling through barrier and inflammatory pathways. Butyrate supports epithelial tight junctions through AMP-activated protein kinase (AMPK)-linked processes (82), whereas impaired barrier integrity facilitates lipopolysaccharide (LPS) translocation (83). LPS can increase epithelial permeability through transforming growth factor-β-activated kinase 1, IκB kinase, and myosin light-chain kinase signaling (84), and peripheral inflammatory information reaches the brain through humoral routes and vagal afferents (85). These pathways connect microbial ecology with the inflammation-oxidative stress loop described above (83–85).

Gut microbial metabolism can also directly shape how neurotransmitter precursors are allocated, with clear relevance to the monoaminergic vulnerability outlined earlier. Most of the body’s serotonin (5-HT) is synthesized in the gut, where gut microbes can promote 5-HT biosynthesis in enterochromaffin cells, defining a well-supported microbiota-regulated peripheral serotonin axis (86). Peripheral 5-HT itself does not cross the blood–brain barrier (BBB) and therefore does not directly supply central serotonergic neurotransmission (87). By contrast, its precursor tryptophan (Trp) can cross the BBB, so peripheral Trp availability constrains the precursor pool available for central 5-HT synthesis (87). The gut microbiota helps determine the metabolic fate of dietary Trp by routing it toward 5-HT synthesis, the kynurenine (Kyn) pathway, or microbial indole derivatives such as indole-3-propionic acid (IPA) (88). Under dysbiosis, immune-linked signaling can bias Trp handling toward Kyn pathway flux and alter indole derivative output, further reducing the effective Trp available for central 5-HT synthesis and plausibly interacting with the inflammation-driven indoleamine 2,3-dioxygenase (IDO) mechanism described earlier (88). Together, these processes reinforce a convergent peripheral-central model that deepens the monoaminergic deficit rather than operating as isolated effects.

Short-chain fatty acids (SCFAs) provide an additional route from microbial metabolism to stress-regulatory circuitry. They signal through free fatty acid receptors 2 and 3 (FFAR2 and FFAR3) on enteroendocrine and neural cells (89), and vagal FFAR3 contributes to propionate-mediated gut-brain communication (90). SCFAs can also modulate vagal signaling and HPA-axis responsiveness (89, 91). Because HPO-HPA coupling changes during perimenopause, variation in SCFA production may modify stress and immune regulation across the transition (18, 36, 89–91). Gut hormones and peripheral metabolic signals, including glucagon-like peptide-1 (GLP-1), peptide YY (PYY), leptin and insulin, also influence brain circuits involved in feeding (92–94)(Figure 3).

### 3.4 Symptom clustering within a multisystem transition

The three axes interact through recurrent feedback loops. Ovarian-hormone variability, stress responsivity, immune-redox tone, sleep disturbance, gut-barrier function, and neurovascular regulation can reinforce one another (3, 4, 20, 66). Sleep disruption, for example, increases HPA and inflammatory signaling, while inflammatory and oxidative processes alter neurotransmitter and neurotrophic pathways involved in mood, cognition, and fatigue (7, 43, 63, 77). SWAN data further show bidirectional associations between vasomotor symptoms and sleep disturbance and identify depression and anxiety as strong predictors of combined vasomotor, sleep, and mood symptoms (95). These observations support a symptom-cluster model of perimenopausal emotional health. This network perspective is well suited to nutritional research because plant-derived exposures often influence several modest biological inputs rather than a single endpoint (96–98). Microbial conversion of isoflavones, lignans, and ellagitannins into equol, enterolignans, and urolithins adds person-specific metabolic variation (99–103). Integrating exposure form, metabotype, symptom pattern, and trial quality therefore provides a practical route from mechanism to clinical study design (3, 4, 104).

## 4 Plant-derived foods, bioactives, and dietary supplements

### 4.1 Plant-rich dietary patterns, whole foods, and exposure hierarchy

This section follows the nutritional exposure continuum from dietary patterns and whole foods to food-derived bioactives, isolated supplements, and standardized botanical preparations. For each exposure, consumed form, regulatory pathways, and human evidence are considered together (Table 1). Plant-rich dietary patterns and whole foods establish the dietary context for bioactive exposure. Mediterranean and related patterns combine fibers, polyphenols, unsaturated fats, minerals, vitamins, fermented foods, and diverse phytochemicals (24, 25). Prospective studies have linked dietary patterns with vasomotor symptoms and later cognitive performance (26, 27), but direct evidence for emotional symptoms in well-staged perimenopausal cohorts remains sparse. Their food matrices influence absorption, microbial metabolism, and downstream biological responses (105–107). Food matrix, co-ingested nutrients, and microbial substrates can alter the bioavailability and metabolite profiles of plant constituents (105–107). Concentrated preparations create higher and more discrete exposures, with pharmacokinetic, interaction, and safety profiles that differ from habitual food intake (105, 108, 109). Reporting both background diet and product formulation is therefore essential in clinical studies.

**Table 1:** Plant-derived foods, bioactives, and dietary supplements by source, exposure category, regulatory axis, and evidence directness.

Bioactive class (examples)	Source and exposure category	Primary axis engaged	Key proposed mechanisms	Microbial metabolite/metabotype	Directness of evidence for perimenopausal emotional health
Soy isoflavones (genistein, daidzein)	Soy foods and legumes (whole food / functional food)	Endocrine-stress; also immune-redox	ERβ-preferential signaling; NF-κB and Nrf2 modulation	Equol (producer status varies)	Menopausal/postmenopausal; VMS relatively robust, mood mixed; no perimenopause-specific mood RCT
Lignans (SDG, enterolignans)	Flaxseed, sesame, and whole grains (whole food)	Endocrine-stress + gut-brain	Estrogen-related and antioxidant pathways; microbial conversion	Enterolignans (enterolactone)	Limited and inconsistent; no robust VMS or mood signal
Curcumin (turmeric)	Turmeric/curry (food source); trials mainly use isolated supplements	Immune-redox-neuroplasticity	NF-κB, NLRP3, Nrf2/ARE; BDNF-CREB and monoaminergic signaling	Extensive metabolism; low bioavailability (piperine, formulations)	Antidepressant meta-analysis in general MDD; not perimenopause-staged
Resveratrol (stilbenoid)	Grapes, berries, and wine (food sources); trials use isolated supplements	Immune-redox-neuroplasticity	SIRT1; mitochondrial and endothelial function; mood/memory in OVX models	Extensive metabolism; variable systemic exposure	Postmenopausal cerebrovascular/cognitive signals; perimenopause unresolved
Tea catechins / anthocyanins (epigallocatechin gallate, EGCG, flavonoids)	Tea, berries, cocoa, fruits, and vegetables (whole-food exposures)	Immune-redox + gut-brain	Antioxidant defense; microglial modulation; microbial metabolite production	Smaller phenolic metabolites	Dietary-pattern level; no isolated perimenopause evidence
L-theanine (tea amino acid)	Tea leaves (food source); trials use isolated L-theanine	Neurotransmitter / stress-inhibitory (GABA/glutamate/monoamine)	GABAergic, glutamatergic, serotonergic/dopaminergic; α-wave activity	Not applicable	Stress and sleep signals in healthy adults; no perimenopause trial
Saffron constituents	Standardized saffron extract (botanical supplement)	Monoaminergic (immune-redox-neuroplasticity)	Serotonergic-related modulation; mood and sleep	Not applicable	Direct perimenopausal RCT on psychological symptoms; strongest direct signal
Ginsenosides (*Panax ginseng* saponins)	Standardized ginseng preparation (botanical supplement)	All three axes (HPA, BDNF, immune-redox, microbiota)	HPA modulation; BDNF-related signaling; anti-inflammatory and redox effects	Microbiota-dependent ginsenoside metabolism; standardization concern	Menopausal/postmenopausal well-being signals; limited, not perimenopause-specific
Product-specific botanical extracts (ERr 731, fenugreek, ashwagandha, shatavari)	Concentrated botanical preparations; not whole-food equivalents	Product-specific endocrine-stress and multi-axis effects	Reported estrogen-related, stress, vasomotor, and psychological-symptom pathways; mechanisms are not interchangeable	Not established	Direct perimenopausal randomized symptom signals, but mostly single-product trials with heterogeneous endpoints
Black cohosh (comparator)	Botanical preparation; menopausal comparator	Thermoregulatory / serotonergic (comparator)	Proposed serotonergic and thermoregulatory actions	Not applicable	Mixed VMS evidence; safety hinges on product identity/standardization; cautionary comparator
Microbiota-active fibers / fermented foods	Legumes, whole grains, fruits, vegetables, and fermented foods (whole food / functional food)	Gut-brain-metabolic	SCFA/butyrate production; barrier integrity; LPS attenuation; gut-brain signaling	SCFA output; urolithin (from ellagitannins) producer status	Gut-brain plausibility; perimenopause-specific emotional-health trials lacking

ARE, antioxidant response element; BDNF, brain-derived neurotrophic factor; CREB, cyclic adenosine monophosphate response element-binding protein; ERβ, estrogen receptor-β; GABA, gamma-aminobutyric acid; HPA, hypothalamic–pituitary–adrenal; LPS, lipopolysaccharide; MDD, major depressive disorder; NF-κB, nuclear factor-κB; NLRP3, NOD-, LRR- and pyrin domain-containing protein 3; Nrf2, nuclear factor erythroid 2-related factor 2; OVX, ovariectomized; RCT, randomized controlled trial; SCFA, short-chain fatty acid; SDG, secoisolariciresinol diglucoside; SIRT1, sirtuin 1; VMS, vasomotor symptoms. ERr 731 denotes a root extract of *Rheum rhaponticum*.

### 4.2 Soy foods, isoflavones, and lignans

Phytoestrogens are prominent plant bioactives in menopausal nutrition because soy foods, legumes, flaxseed, sesame, and related foods provide recognizable dietary sources (110–112). Their phenolic structures permit tissue-, receptor-, and concentration-dependent interaction with estrogen signaling (110). This interaction is particularly relevant during perimenopause, when ovarian hormones fluctuate substantially (13–15). Estrogen-receptor engagement depends on molecular structure. Genistein and daidzein have near-planar diphenolic ring systems whose hydroxyl spacing resembles that of 17β-estradiol, allowing them to occupy the estrogen-receptor ligand-binding pocket and contributing to their preference for estrogen receptor-β (ERβ) over estrogen receptor-α (ERα) (110). Lignans and a limited group of related phenolics share aspects of this structural logic. Most dietary polyphenols lack the same hydroxyl geometry and are better examined through inflammatory, redox, or microbial-metabolite signaling. Estrogenic activity cannot therefore be inferred from the umbrella term polyphenol (Figure 4). Soy isoflavones such as genistein and daidzein have been examined across vascular, thermoregulatory, psychological, and cognitive outcomes in menopausal populations and show preferential affinity for ERβ (110, 112, 113). Experimental studies also link isoflavones with NF-κB- and Nrf2-related regulation, connecting endocrine signaling with the immune-redox-neuroplasticity axis (114–117).

**Figure 4:**
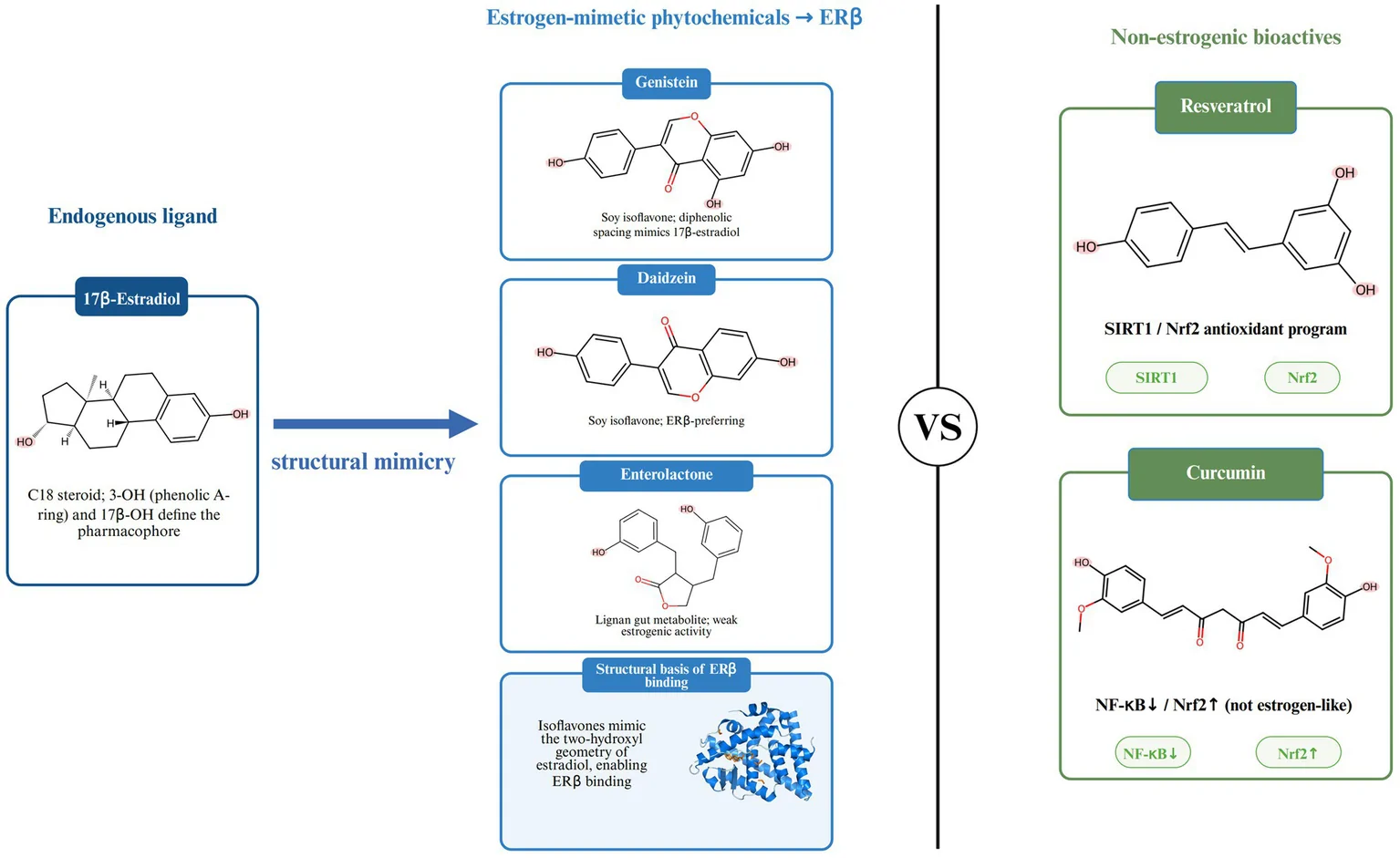
Structural similarity between representative food-derived bioactives or isolated supplements and 17β-estradiol, and their molecular targets. Estrogen-mimetic phytochemicals (genistein, daidzein, enterolactone) share the diphenolic hydroxyl geometry of 17β-estradiol, favoring estrogen receptor-β (ERβ), whereas bioactives acting predominantly through redox and neuroplasticity targets (resveratrol, curcumin) engage Nrf2, NF-κB and SIRT1 signaling, with resveratrol additionally reported to interact with estrogen-related pathways. Table 1 specifies the exposure form and evidence level represented by each class. NF-κB, nuclear factor-κB; Nrf2, nuclear factor erythroid 2-related factor 2; SIRT1, sirtuin 1. (Created by BioRender.com).

One central source of heterogeneous response lies in microbial metabolism, which anchors soy isoflavones to the gut-brain-metabolic axis. Specific gut bacteria can convert daidzein into equol, a metabolite with stronger estrogen-related activity than its precursor, yet the capacity to produce equol varies substantially across individuals (99, 100, 118, 119). Trial and review data indicate that equol-producer status may modify responses to soy isoflavones, although findings are not uniform (120–122). Equol-producer status therefore warrants consideration in future perimenopause trials as a candidate effect modifier rather than a peripheral methodological detail. Lignans from flaxseed, sesame and whole grains constitute a second, microbiome-dependent phytoestrogen route that similarly converges on the gut-brain-metabolic axis. Secoisolariciresinol diglucoside and related lignans are converted by intestinal microbes into enterolignans, which can engage estrogen-related and antioxidant pathways (98, 101, 111). The supporting evidence remains more limited and less consistent than the isoflavone literature; flaxseed trial data do not yet support a robust vasomotor-symptom effect, but lignans remain biologically relevant because of their microbiota-dependent conversion and estrogen-related or antioxidant pathways (98, 111, 123, 124). Clinical responses to phytoestrogens vary with reproductive stage, baseline diet, dose, food matrix, and microbial metabotype. The largest clinical literature concerns vasomotor and broader menopausal symptoms (112, 125–127), while psychological outcomes are less extensively studied and more heterogeneous (128–130). These features make phytoestrogens a useful model for integrating food form, endocrine signaling, and microbiome-dependent response.

### 4.3 Polyphenol-rich foods and isolated supplements

Polyphenols and flavonoids broaden the food-level evidence because they occur throughout fruits, vegetables, tea, cocoa, berries, herbs, and spices (96, 97, 131, 132). Their chemical diversity produces distinct biological profiles. Many influence inflammatory and oxidative pathways (96, 133), and microbial metabolism generates additional compounds that participate in systemic and gut-brain signaling (134, 135). The following examples are therefore considered individually and within their food or formulation context. Curcumin, the principal curcuminoid in turmeric, engages NF-κB, NLRP3, and Nrf2/ARE signaling in experimental systems (133, 136–138). Ovariectomy and stress models also connect curcumin with monoaminergic signaling and neuroplasticity involving BDNF and cAMP response element-binding protein (CREB) (139–142). Meta-analyses report antidepressant effects in adults with major depressive disorder (143, 144), providing adjacent human evidence for future perimenopause-specific evaluation.

Resveratrol, a stilbenoid found in grape skins, berries, and related foods, has been studied for sirtuin 1 (SIRT1)-linked inflammatory regulation, mitochondrial function, and endothelial biology (145–147). Menopause models extend these pathways to mood and memory after ovarian-hormone loss (148, 149), and postmenopausal trials report cerebrovascular and cognitive outcomes (150). Perimenopause-specific emotional outcomes remain an open clinical question. Epigallocatechin gallate and quercetin modulate antioxidant and inflammatory responses in microglial cell models (151, 152). Anthocyanins have been detected in the brains of aged blueberry-fed rats (153), and higher berry intake has been associated with slower cognitive decline in older women (154). Current human evidence supports their evaluation mainly as constituents of plant-rich dietary patterns and polyphenol-rich foods (96, 97, 155, 156).

### 4.4 Tea-derived compounds and botanical preparations

L-theanine is a non-proteinogenic amino acid from tea that has been studied for stress, anxiety, and sleep outcomes. Proposed actions involve GABAergic, glutamatergic, serotonergic, dopaminergic, and α-wave-associated pathways (157–160). These mechanisms overlap with stress-GABA regulation during perimenopause and provide a basis for dedicated trials in this population. The remaining candidates in this subsection are standardized botanical preparations. Randomized perimenopausal trials have evaluated saffron, rhapontic rhubarb ERr 731, fenugreek, ashwagandha, and shatavari extracts (28–32). Ginseng and valerian have mainly been studied in adjacent populations, and black cohosh provides a comparator from menopausal botanical research (161, 162). Each preparation has a distinct composition, dose, standardization process, and regulatory context.

A randomized, double-blind, placebo-controlled trial of standardized saffron extract in perimenopausal women reported improvements in psychological symptoms and negative affect, without corresponding superiority for vasomotor or somatic symptoms (28). The product-specific result provides direct symptom evidence and a clear target for independent replication with monoaminergic, inflammatory, and neurotrophic biomarkers. Beyond saffron, ERr 731 rhapontic rhubarb reduced anxiety and improved well-being; fenugreek and ashwagandha improved psychological items or domains within menopause scales; and shatavari improved menopausal symptoms, stress, and selected fatigue and vigor measures (29–32). These studies used different preparations and outcome hierarchies, whereas the Hypericum-Vitex trial found no superiority over placebo (33). The comparison identifies product-specific psychological outcomes for independent replication rather than a common botanical effect (Table 2).

**Table 2:** Representative direct and comparator clinical evidence for plant-derived foods, bioactives, and dietary supplements.

Exposure / preparation	Population, stage (design, sample size)	Dose / duration	Key finding	Directness	Quality notes
Standardized saffron extract (affron®) (28)	Perimenopausal, 40–60 y (double-blind RCT, *n* = 86; 82 analyzed)	14 mg twice daily (28 mg/d), 12 wk	Improved psychological score and negative affect; no superiority for vasomotor or somatic outcomes	Direct; one cited product-specific trial	Mood secondary; 11.6% withdrawal; industry-funded; author ties disclosed.
Rhapontic rhubarb extract (ERr 731; constituents unquantified) (29)	Perimenopausal with climacteric complaints and anxiety (double-blind RCT; 110 randomized, 109 in intention-to-treat analysis)	One enteric-coated tablet daily, 12 wk	Reduced Hamilton Anxiety scores and improved well-being	Direct; replication needed	Anxiety/well-being exploratory secondary; completers 39/54 vs. 7/55; manufacturer-commissioned; sponsor/contract-research author affiliations.
Standardized fenugreek seed extract (30)	Perimenopausal with MRS-defined symptoms (double-blind RCT, *n* = 48)	250 mg twice daily, 42 d	Improved selected MRS domains, including depression and insomnia symptom items	Direct; small secondary-domain signal	Depression was a scale item; 12.5% withdrawal; industry-funded; author ties disclosed.
Standardized ashwagandha root extract (31)	Perimenopausal with climacteric symptoms (double-blind RCT, *n* = 100; 91 completed)	300 mg twice daily, 8 wk	Reduced total and psychological MRS domains; emotional outcomes were embedded in a broad symptom scale	Direct; one cited product-specific trial	Psychological domain within primary MRS; 9% noncompletion; supplier-provided products; funding unstated; authors declared no conflicts.
Standardized shatavari root extract (32)	Perimenopausal with symptoms (double-blind RCT, *n* = 80; 73 completed)	300 mg/d, 8 wk	Improved MRS and stress scores, with selected fatigue and vigor signals	Direct; one cited product-specific trial	Mood/stress secondary; 8.8% attrition; supplier-provided products; funding unstated; authors declared no conflicts.
Hypericum-Vitex combination (Hypericum standardized; Vitex unstandardized) (33)	Late perimenopausal or postmenopausal (double-blind RCT, *n* = 100; 93 completed)	Combination tablets twice daily, 16 wk	No superiority for hot flashes, Hamilton Depression, climacteric symptoms, or quality of life	Direct/adjacent; null trial	Depression secondary; 7% noncompletion; college/foundation-funded; supplier-provided products; author ties disclosed.
Rosa damascena extract capsules (phenolic-content standardized) (189)	Naturally postmenopausal, 45–60 y (triple-blind RCT, *n* = 82)	500 mg twice daily, 8 wk	Reduced DASS-21 depression, anxiety, and stress scores	Adjacent; postmenopausal	DASS-21 secondary in registry; 6.1% noncompletion; no specific grant; university support; authors declared no conflicts.
Isolated curcumin supplements (varied formulations) (144)	Adults with depression or MDD (meta-analysis, 9 reports; 10 comparisons; *n* = 531)	Varied by trial, 4–12 wk	Antidepressant signal (Hedges’ g = −0.75)	Broader-adult	Depression primary; trial-level attrition; review declared no specific funding or conflicts.
Isolated L-theanine (157)	Healthy adults, mean age about 48 y (crossover RCT, *n* = 30)	200 mg/d, 4 wk	Reduced stress, anxiety, and sleep scores	Broader-adult	Symptoms/cognition primary; no attrition; industry-funded; employee authors; symptom benefit over placebo limited to selected sleep subscales.
Soy isoflavone aglycone supplement (composition quantified) (129)	Menopausal women, 40–60 y (double-blind RCT, *n* = 90)	12.5 or 25 mg/d, 8 wk	Low-dose group showed improvement in selected psychological symptoms	Adjacent; menopausal	No single primary endpoint specified; 3.3% attrition; industry-funded; author research grant disclosed.

Quality notes summarize selected study features; no formal risk-of-bias assessment was performed. MRS, Menopause Rating Scale; DASS-21, 21-item Depression Anxiety Stress Scales; RCT, randomized controlled trial; MDD, major depressive disorder.

Ginsenosides from *Panax ginseng* influence HPA and BDNF-related signaling (163–167), together with inflammatory, redox, and microbiota-linked pathways that span all three regulatory axes (168–171). Human studies in menopausal and postmenopausal women report signals in psychological distress, fatigue, and general well-being (161, 172, 173). Interpretation remains product-dependent and requires adequate reporting and quality control (174, 175). Black cohosh preparations have been studied primarily for vasomotor symptoms and may involve serotonergic and thermoregulatory pathways (62, 176–178). Findings vary by extract, and interpretation depends on product identity, standardization, dose, duration, and hepatic safety monitoring. These characteristics make black cohosh an informative comparator for the quality-control challenges of botanical products.

### 4.5 Fibers, fermented foods, food matrix, and microbiota

Microbiota-active dietary components connect plant-rich diets with the gut-brain-metabolic axis. Reproductive aging is accompanied by changes in the microbiome and estrobolome (78, 81), while short-chain fatty acids, tryptophan metabolites, and barrier-related signals shape immune and neural communication (82, 88, 89, 156). These pathways provide measurable targets for studies of mood, sleep, fatigue, and cognition during perimenopause. Dietary fibers, resistant starches, inulin-type fructans, legumes, whole grains, fruits, and vegetables support microbial fermentation and short-chain fatty acid production (155, 179). Butyrate and related metabolites preserve epithelial barrier integrity and attenuate inflammatory signaling associated with LPS translocation (82–85). Biomarker-rich dietary trials can test whether these effects translate to emotional and fatigue-related outcomes during the menopausal transition.

Polyphenols are similarly microbiota-active. Ellagitannins from pomegranate, berries, and nuts can be biotransformed into urolithins (102, 103), whereas tea catechins, curcumin, and other polyphenols may remodel microbial communities and yield smaller phenolic metabolites with systemic activity (134, 135, 180–182). The marked interindividual variability in equol, urolithin, and enterolignan production further provides a strong rationale for metabotype-aware trial design rather than one-size-fits-all supplementation (99, 101–103). Probiotics, prebiotics, synbiotics, and fermented foods extend this framework to defined strains, substrates, and food matrices (183–185). Their broader mood and sleep literature supports perimenopause-specific studies that combine emotional outcomes with microbiome, metabolite, and barrier-function endpoints.

Melatonin also occurs in plant foods, including cherries, grapes, tomatoes, and nuts (186). Small cherry-juice trials have reported improvements in selected sleep measures (187), but these studies do not isolate the effects of melatonin from those of other food constituents. Dietary exposure differs in dose and food matrix from isolated melatonin supplements, commonly synthetic, and from endogenous circadian secretion (186). Findings from supplementation cannot therefore establish the effects of melatonin-containing foods on perimenopausal emotional health. Within this review’s framework, food-derived melatonin is best regarded as a circadian-relevant component of plant-rich dietary patterns whose intake could be recorded in future perimenopausal nutrition trials, rather than as a candidate intervention.

### 4.6 Hypotheses linking symptom profiles to candidate exposures

Symptom clustering provides a bridge between the three-axis framework and prospective trial design. Mood, vasomotor, sleep, and fatigue symptoms occur in different combinations across the menopausal transition (3, 4, 95). Relating symptom patterns to proposed exposure-related pathways can inform hypotheses about cohort selection, endpoint choice, and exploratory biomarker measurement. Proposed mechanistic links include endocrine-stress, monoaminergic, and GABAergic regulation in mood-predominant presentations; thermoregulatory, noradrenergic, and sleep-related neuroimmune processes in combined vasomotor-sleep-mood presentations; and immune-redox and gut-brain-metabolic processes in fatigue-predominant presentations (3, 4, 95). Candidate exposures also differ in pathway coverage. Saffron has direct symptom evidence and proposed monoaminergic actions (28); L-theanine centers on stress-GABA mechanisms (157–159); phytoestrogens engage endocrine-stress pathways (110–112); curcumin spans neuroimmune and neuroplasticity pathways (133, 136–138, 144); ginsenosides engage HPA, BDNF, redox, and microbiota-linked mechanisms (164, 168–171); and microbiota-active fibers primarily engage the gut-brain-metabolic axis (179, 183).

These patterns yield three trial hypotheses: standardized saffron or isolated L-theanine for mood-predominant cohorts; ginseng preparations or phytoestrogen exposures for cohorts combining vasomotor, sleep, and mood symptoms; and isolated curcumin or microbiota-active dietary strategies for fatigue-predominant cohorts. Each design can prespecify the relevant emotional endpoint and include biomarkers from the pathways predicted to respond (Table 3). Evidence directness varies across these mappings. Saffron provides a direct perimenopausal symptom signal, although the trial did not measure target engagement (28). ERr 731, fenugreek, ashwagandha, and shatavari preparations have also produced psychological-domain signals in perimenopausal trials (29–32). Evidence for L-theanine, ginseng, phytoestrogens, curcumin, and microbiota-active strategies comes mainly from adjacent populations or mechanistic studies. These mappings are hypothesis-generating rather than prioritized conclusions. Prospective phenotype-stratified trials are needed to test whether baseline symptom profiles modify intervention response; mechanistic plausibility or biomarker change alone does not validate the proposed matching.

**Table 3:** Hypothesis-generating links between symptom profiles and candidate exposures for prospective evaluation.

Symptom presentation	Proposed mechanistic rationale	Candidate exposure(s) for prospective evaluation	Evidence and limitations
Mood-predominant	Endocrine-stress; monoaminergic / GABAergic	Standardized saffron extract; isolated L-theanine	Saffron: direct; one cited product-specific trial; target engagement unassessed; replication needed (28). L-theanine: broader-adult trials; perimenopausal applicability uncertain (157, 158).
Combined vasomotor-sleep-mood	All three axes; thermoregulatory, noradrenergic, sleep-related neuroimmune	Ginseng preparation; soy foods or isoflavone supplement	Adjacent menopausal/postmenopausal evidence; preparation- and outcome-dependent (128–130, 161, 173); supplement findings not directly transferable to soy foods.
Fatigue-predominant (somatic-psychological)	Immune-redox; gut-brain-metabolic	Isolated curcumin; microbiota-active dietary strategies	Indirect clinical/preclinical evidence; heterogeneous preparations and outcomes; fatigue-specific perimenopausal efficacy unestablished (139, 140, 144, 179, 183).

Evidence categories indicate population directness, not formal certainty. All links are hypothesis-generating; phenotype-specific benefit and the proposed mechanisms remain unvalidated. GABA, gamma-aminobutyric acid.

## 5 Clinical evidence and its interpretation

### 5.1 Emotional health, sleep, and cognitive outcomes

Clinical evidence is organized below by outcome domain and exposure form, with reproductive stage and study design incorporated into each interpretation. Direct perimenopausal trials are considered alongside adjacent menopausal or postmenopausal studies and broader-adult evidence to show where clinical signals are most developed and where dedicated trials are needed. Vasomotor, sleep, and cognitive outcomes help interpret emotional health because these domains frequently cluster during perimenopause. The largest menopause-related evidence base concerns vasomotor symptoms and includes phytoestrogens, red clover, flaxseed, black cohosh, and other nonhormonal approaches (104, 125–127). Findings vary with menopausal stage, symptom severity, dose, preparation, duration, and equol-producer status (112, 122, 126). A 2026 study of 2,097 postmenopausal women also associated recalled perimenopausal sleep disturbance with lower later-life cognitive scores, particularly when sleep was disrupted by menopausal symptoms (188). These linked domains help define clinically coherent symptom clusters for nutrition trials.

Across direct perimenopausal trials, randomized samples ranged from 48 to 110 participants and follow-up from 42 days to 12 weeks (Table 2) (28–32). Emotional outcomes ranged from dedicated anxiety or affect measures to psychological domains and individual symptom items within broader menopause scales. Because preparations and outcome hierarchies differed, these studies are best interpreted as product-specific signals that define designs for independent replication. Results across other exposures are heterogeneous. Hypericum plus Vitex showed no advantage over placebo for Hamilton Depression scores or other prespecified outcomes (33), whereas Rosa damascena capsules reduced depression, anxiety, and stress in postmenopausal women (189). Curcumin has meta-analytic support in major depressive disorder (144); soy-isoflavone trials report mixed psychological outcomes in menopausal samples (128–130); and L-theanine and ginseng evidence remains broader-adult or product-dependent (157, 161). This diversity favors exposure-specific evaluation rather than broad botanical grouping.

Sleep disruption interacts closely with mood and stress regulation during perimenopause. Valerian has been studied in menopausal and postmenopausal sleep complaints (162, 190), L-theanine in stressed adults (157), and saffron in sleep-quality and perimenopausal-symptom contexts (28, 191). Objective sleep architecture is rarely measured, highlighting the value of perimenopause-specific trials that combine actigraphy or polysomnography with emotional outcomes. Cognitive outcomes add a further dimension to the sleep-mood-fatigue cluster. Soy-isoflavone trials and reviews report variable effects across selected cognitive domains, with age, intervention timing, baseline status, and outcome measure as potential modifiers (192–194). Resveratrol trials in postmenopausal women report cerebrovascular and cognitive outcomes (150), and Mediterranean-diet adherence has been associated with later semantic verbal fluency (27). These findings support integrated studies of cognition, sleep, and emotional health during the transition.

### 5.2 Food matrix, preparation, bioavailability, and response heterogeneity

Bioavailability links food or product intake with biological response. Curcumin, resveratrol, isoflavones, lignans, and many flavonoids undergo extensive intestinal, hepatic, and microbial metabolism, and their circulating metabolites can differ markedly from the parent compounds studied *in vitro* (105, 195, 196). Microbial and hepatic transformations contribute to substantial interindividual variation in systemic exposure (106, 107, 197, 198). Human trials can address this variation by measuring circulating metabolites and pathway-specific target engagement. Food matrix and formulation shape both exposure and interpretation. Whole foods provide repeated, lower-dose mixtures, whereas concentrated extracts produce distinct pharmacokinetic and safety profiles (105, 108, 109). In an early pharmacokinetic study, piperine increased curcumin exposure (199). Phospholipid complexes, nanoparticles, and amorphous dispersions further alter delivery (200–202). Recording matrix, formulation, dose, and background diet allows clinical findings to be assigned to the exposure actually tested.

Where feasible, future studies should incorporate exposure and target-engagement markers, including equol status, urolithin metabotype, enterolignan production and circulating polyphenol metabolites (99, 101–103). Mechanism-oriented endpoints could encompass inflammatory and oxidative stress markers (203, 204), BDNF-related readouts where clinically justified (76, 77), cortisol rhythms (205), vascular indices and microbiome-related endpoints. Such measurements would help distinguish nonresponse rooted in biology from nonresponse attributable to inadequate exposure.

### 5.3 Priorities for perimenopause-specific clinical trials

The Stages of Reproductive Aging Workshop + 10 (STRAW+10) provides consensus criteria for staging reproductive aging, based primarily on menstrual bleeding patterns, with biomarkers serving as supportive rather than required criteria (206). Reproductive staging should be incorporated into trial design because late reproductive, early-transition, late-transition, and early-postmenopausal stages differ in endocrine variability, symptom pattern, and sleep disturbance (7, 13, 14, 206). A STRAW+10-based analysis found incremental worsening of sleep quality across six reproductive-aging substages independent of chronological age, whereas a three-stage classification was less informative (207). Stage-stratified recruitment and analysis can therefore reveal response patterns that pooled samples may miss. Biological timing may influence neurovascular and neural responses to intervention, as shown in hormone-therapy and cognition research (208–210). Perimenopause offers a defined period in which endocrine, neural, and metabolic systems are actively remodeling. Prospective studies initiated at specific STRAW+10 stages can test whether nutritional response varies across this window.

Future trials should integrate STRAW+10 staging (206), symptom-cluster phenotyping (3, 4), dietary-context assessment and microbiome/metabotype profiling (78, 81, 100, 101, 156), and validated outcome instruments for sleep, mood, cognition, vasomotor symptoms, fatigue and quality of life. Layering mechanism-oriented endpoints onto this design, such as salivary cortisol rhythms (205), inflammatory and oxidative stress markers (203, 204), BDNF (76, 77), endothelial function and microbiome-derived metabolites, would support exploratory assessment of biological responses. The proposed symptom–exposure hypotheses require prospective evaluation (Table 3). Trials should define symptom profiles in advance and test whether they modify intervention response; mechanistic plausibility alone does not establish which exposure is preferable for a given profile.

### 5.4 Safety, product quality, and clinical implications

Safety depends on intake range, formulation, comorbidities, and concomitant medication. Systematic reviews and large observational datasets on soy and isoflavones generally provide reassurance across breast, endometrial, and other hormone-sensitive outcomes when exposure is interpreted in context (211–214). Concentrated extracts require product-level evaluation. Curcumin-piperine products can interact with platelet pathways and drug-metabolizing enzymes (215, 216), while resveratrol and black cohosh require preparation-specific safety assessment (177, 178, 217). Enhanced formulations and variable supplement quality can also produce exposures that differ from ordinary diets (105, 109, 174, 175). Dose–response relationships remain incompletely characterized in perimenopausal populations. Habitual foods provide sustained, low-level, multi-compound exposure, whereas supplements can achieve higher concentrations and measurable biomarker changes (105, 109). Trials that compare food-based and supplemental delivery can clarify the exposure required for pathway engagement and identify corresponding safety thresholds.

Interactions with prescribed medicines are a further consideration, because women with perimenopausal mood symptoms may already be receiving antidepressants. St John’s wort (*Hypericum perforatum*) can induce cytochrome P450 enzymes, including CYP3A4 and CYP2C9, and P-glycoprotein, with induction influenced by the preparation’s hyperforin content and dose. These pharmacokinetic interactions may reduce the exposure or effectiveness of concomitant medicines, including oral contraceptives, cyclosporine, and warfarin (218). Concomitant use with selective serotonin reuptake inhibitors (SSRIs) may produce additive serotonergic effects and increase the risk of serotonin syndrome; this combination should be avoided (219). Concomitant prescription medicines and herbal products should therefore be reviewed before St John’s wort is started (218, 219).

Clinical interpretation should separate general dietary guidance from product-specific treatment evidence. Plant-rich dietary patterns support general menopausal and cardiometabolic health (24, 25), while meta-analyses of emotional outcomes and plant-derived supplements report heterogeneous interventions and variable study quality (34, 35). The 2023 North American Menopause Society position statement does not recommend supplements, herbal remedies, soy foods or extracts, equol, or dietary modification as evidence-based treatments for vasomotor symptoms (104). For emotional health, existing perimenopausal trials are small and product-specific (28–32). Current evidence is therefore insufficient to recommend a specific plant-derived supplement as an established treatment for perimenopausal depression, anxiety, or insomnia. Severe or persistent symptoms require individualized clinical assessment.

## 6 Research priorities for nutritional translation

### 6.1 Metabotypes and precision-nutrition stratification

Future research should move beyond compound inventories toward studies grounded in reproductive stage, mechanism, and dietary context. Integrating reproductive stage, symptom phenotype, exposure biomarkers, microbiome function, and patient-relevant outcomes will make nutritional trials more informative and clinically interpretable. Precision nutrition is pertinent because response to plant bioactives is shaped by host metabolism, microbiome function, baseline diet, body composition, medication use, and symptom phenotype. Metabotyping and metabolomics provide tools for characterizing this variation (220, 221), and gut-microbiome features can modify responses to dietary interventions (222, 223). Equol, urolithin, and enterolignan production illustrates how microbial metabotypes alter exposure to soy isoflavones, ellagitannins, and lignans (99, 101–103). Trials can test whether equol-producer status, urolithin metabotype, diet quality, inflammatory profile, sleep phenotype, or cortisol rhythm improves prediction of response.

### 6.2 Study designs for diet-brain-hormone interactions

N-of-1 and pragmatic trial frameworks can capture individual response variability in complex nutrition interventions (224, 225). Longer follow-up with serial microbiome and biomarker assessment can determine whether polyphenol-rich interventions alter microbial, inflammatory, and oxidative-stress pathways (226). Systems biology and multi-omics can map interacting mechanisms (227–229), while exposure-metabolome methods can improve measurement of polyphenol intake and metabolism (230). Combining these approaches with prospective clinical endpoints will clarify which molecular signatures accompany meaningful symptom change.

### 6.3 Functional foods and culturally grounded dietary models

Functional foods provide a practical route for translating plant-bioactive research into nutrition strategies. Product development should emphasize recognizable food matrices, transparent composition, feasible intake patterns, and compatibility with habitual diets (231). Culturally grounded interventions should also specify how a product is incorporated into familiar meals, whether it replaces or adds to usual foods, and how processing changes the delivered bioactive dose. Food-matrix effects on bioavailability require direct assessment (106, 107), and fermented foods and soy products illustrate how microbial processing reshapes bioactive exposure along the gut-brain-metabolic axis (184, 232, 233). Batch-level quality control, safety monitoring, and clear labeling should accompany formulation from the outset (174, 175).

## 7 Conclusion

Perimenopause links ovarian-hormone variability with changes in stress physiology, immune-redox balance, neuroplasticity, and gut-brain metabolism. These interacting systems help explain why depressive symptoms, anxiety, sleep disturbance, vasomotor symptoms, fatigue, and cognitive complaints often cluster during the transition. Plant-derived nutrition spans habitual dietary patterns, whole foods, isolated food-derived bioactives, dietary supplements, and standardized botanical preparations. Food matrix, dose, metabolism, product standardization, and reproductive stage all shape biological exposure and clinical response. Organizing the literature across the endocrine-stress, immune-redox-neuroplasticity, and gut-brain-metabolic axes connects these nutritional features with the biology of emotional health.

Direct perimenopausal trials report product-specific psychological-symptom signals for standardized saffron, rhapontic rhubarb, fenugreek, ashwagandha, and shatavari preparations, alongside a null Hypericum-Vitex trial. Evidence for dietary patterns, isolated bioactives, and other supplements derives mainly from adjacent menopausal, broader-adult, or mechanistic studies. Current evidence is insufficient to recommend a specific supplement as an established treatment, and the proposed symptom–exposure mappings remain hypothesis-generating. Future phenotype-stratified trials should integrate STRAW+10 staging, emotional-health phenotyping, baseline diet, food matrix, microbial metabotypes, exposure biomarkers, standardized preparation characterization, and safety monitoring.
